# The interplay between sex hormones, mitochondrial dysfunction, and intervertebral disc degeneration: insights beyond Qiu et al.

**DOI:** 10.1186/s12967-024-06026-5

**Published:** 2024-12-31

**Authors:** Yu-Jen Lu, Hong Kai Wang, Yu-Hsiang Lin

**Affiliations:** 1 Department of Neurosurgery, Chang Gung Memorial Hospital at Linkou, 5 Fu-Shing Street, Kweishan, Taoyuan, 333 Taiwan; 2 School of Medicine, Chang Gung University, Kwei-shan, Tao-Yuan, Taiwan; 3 Department of Urology, Chang Gung Memorial Hospital at Linkou, 5 Fu-Shing Street, Kweishan, Taoyuan, 333 Taiwan

Dear Editor,

We read with great interest the recent work by Qiu et al. [1], which provided a comprehensive exploration of the relationship between mitochondrial quality control (MQC) and intervertebral disc degeneration (IVDD). The authors convincingly outlined the pivotal role of mitochondrial dysfunction in IVDD, highlighting mechanisms such as energy imbalance, apoptosis, oxidative stress, and dysregulated extracellular matrix metabolism. These insights offer a promising foundation for understanding IVDD pathogenesis.

Building upon their findings, we propose additional considerations regarding the role of sex hormones in mitochondrial health and IVDD progression. As demonstrated in prior studies, testosterone levels decline with aging in men [2], and this reduction exacerbates mitochondrial dysfunction, leading to oxidative stress and impaired cellular energy homeostasis [3]. Qiu et al. touched upon oxidative stress and energy imbalance, but integrating the hormonal dimension could deepen our understanding of the pathophysiology.

Notably, age-related testosterone decline creates a vicious cycle: Benign prostatic hyperplasia (BPH), common in aging men, exacerbates nocturia and disrupts circadian rhythms and the hypothalamic-pituitary-gonadal (HPG) axis [4]. These disruptions further suppress testosterone production, perpetuating mitochondrial dysfunction and exacerbating IVDD.

Moreover, postmenopausal women experience a loss of estrogen, another critical sex hormone with known protective effects on mitochondrial function [5]. Estrogen modulates mitochondrial dynamics, biogenesis, and oxidative stress, suggesting that its deficiency could similarly aggravate IVDD through impaired MQC.

Given the interconnection between sex hormones and mitochondrial function, we believe future IVDD therapies should explore hormone supplementation as an adjunct strategy. Targeted interventions aimed at restoring mitochondrial health via sex hormone modulation could mitigate IVDD progression in both men and women.

We commend Qiu et al. for their valuable contribution and encourage further investigation into this multidimensional interplay of hormones, mitochondria, and IVDD.

## Data Availability

Not applicable.
